# Ginsenoside Rh1 Improves the Effect of Dexamethasone on Autoantibodies Production and Lymphoproliferation in MRL/lpr Mice

**DOI:** 10.1155/2015/727650

**Published:** 2015-03-31

**Authors:** Yinglu Feng, Chunbin Wang, Silu Cheng, Xiaorong Wang, Xianze Meng, Lujia Li, Juan Du, Qun Liu, Yuyu Guo, Yongbin Meng, Binbin Cheng, Changquan Ling

**Affiliations:** ^1^Department of Traditional Chinese Medicine, 401 Hospital of the Chinese People's Liberation Army, Qingdao, Shandong 266071, China; ^2^Department of Traditional Chinese Medicine, Changhai Hospital, Second Military Medical University, Shanghai 200433, China; ^3^Department of Oncology, 534 Hospital of the Chinese People's Liberation Army, Luoyang, Henan 471003, China; ^4^Department of Oncology, Suzhou Hospital of Traditional Chinese Medicine, Suzhou, Jiangsu 215009, China; ^5^E-Institute of TCM Internal Medicine, Shanghai Municipal Education Commission, Shanghai 201203, China

## Abstract

Ginsenoside Rh1 is able to upregulate glucocorticoid receptor (GR) level, suggesting Rh1 may improve glucocorticoid efficacy in hormone-dependent diseases. Therefore, we investigated whether Rh1 could enhance the effect of dexamethasone (Dex) in the treatment of MRL/lpr mice. MRL/lpr mice were treated with vehicle, Dex, Rh1, or Dex + Rh1 for 4 weeks. Dex significantly reduced the proteinuria and anti-dsDNA and anti-ANA autoantibodies. The levels of proteinuria and anti-dsDNA and anti-ANA autoantibodies were further decreased in Dex + Rh1 group. Dex, Rh1, or Dex + Rh1 did not alter the proportion of CD4+ splenic lymphocytes, whereas the proportion of CD8+ splenic lymphocytes was significantly increased in Dex and Dex + Rh1 groups. Dex + Rh1 significantly decreased the ratio of CD4+/CD8+ splenic lymphocytes compared with control. Con A-induced CD4+ splenic lymphocytes proliferation was increased in Dex-treated mice and was inhibited in Dex + Rh1-treated mice. Th1 cytokine IFN-*γ* mRNA was suppressed and Th2 cytokine IL-4 mRNA was increased by Dex. The effect of Dex on IFN-*γ* and IL-4 mRNA was enhanced by Rh1. In conclusion, our data suggest that Rh1 may enhance the effect of Dex in the treatment of MRL/lpr mice through regulating CD4+ T cells activation and Th1/Th2 balance.

## 1. Introduction

Systemic lupus erythematosus (SLE), a chronic autoimmune disease, is characterized by the presence of autoantibodies and the deposition of immune complexes in multiple organs, including the skin, kidneys, heart, and joints, resulting in inflammation and tissue damage [1, 2]. To date, glucocorticoids (GCs) are still the first-line drugs for SLE. Although most patients with active SLE benefit from the anti-inflammatory action of GCs, approximately 30% of SLE patients do not respond sufficiently and thus require a higher dose [3]. However, high dose and/or prolonged GCs administration usually causes severe side effects, such as osteoporosis, cardiovascular events, diabetes mellitus, infection, and hemorrhage of digestive tract that seriously affect the quality of life and the prognosis of patients [4].

The reasons for GC insensitivity have been widely reported previously, such as decreased expression of glucocorticoid receptor alpha (GR*α*), which is a member of the nuclear receptor family of ligand-dependent transcription factors and mediates most GCs actions, reduced DNA-binding ability of GR*α*, increased GRbeta (*β*) transcript levels, and enhanced expression of inflammatory transcription factors like AP-1 or NF*κ*B [5–8]. It is widely recognized that the level of GR*α* expression is closely correlated with GCs response [9]. However, administration of GR*α* agonists usually downregulates GR*α* levels and thereby limits the therapeutic responses to GCs. In contrast, upregulation of GR*α* is associated with enhanced glucocorticoid sensitivity. Therefore, agents that may upregulate the expression of GR*α* in the process of GC treatment would enhance the efficacy of GCs in SLE patients and thereby reduce the GCs dose and side effects.

In the past decade, our group continuously focused on the regulation of GR with Traditional Chinese Medicine [10, 11]. Ginsenosides (GSS), the main extracts of ginseng, are able to partially reverse dexamethasone (Dex) induced downregulation of GR expression and hormone binding activity in HL7702 cells and subsequently enhance Dex induced transcription of reporter gene [12]. This effect was also confirmed in rats [10]. Among the numerous ingredients of GSS, Rh1 has been identified as an effective part accounting for the GR upregulation after Dex treatment and enhances the anti-inflammatory effect of Dex in collagen-induced arthritis (CIA) mouse model [13]. This exciting result suggests Rh1 may be also effective in improving GCs efficacy in other hormone-dependent diseases. Therefore, we further investigated the effect of Rh1 in combination with Dex in the treatment of SLE mouse model.

## 2. Materials and Methods

### 2.1. Animals

Eight-week old female MRL/lpr mice were purchased from Shanghai Laboratory Animal Center and maintained in standard animal cages under specific pathogen-free conditions in the Laboratory Animal Center of the Second Military Medical University with the dark/light cycle of 12 h at 22°C. The animals were maintained with food and water available ad libitum and housed for a week prior to the experiment. The experiments were performed in accordance with the European Communities Council Directive of 24 November 1986 (86/609 EEC) and approved by the Ethics Committee of Changhai Hospital.

### 2.2. Drug Administration

Twenty-four female MRL/lpr mice were randomly divided into 4 groups: (1) control group; (2) Dex group; (3) Rh1 group; (4) Dex + Rh1 group. Mice were intraperitoneally injected with 10% ethanol (vehicle for Rh1 and Dex), Dex (1 mg·kg^−1^·d^−1^), Rh1 (25 mg·kg^−1^·d^−1^), or Dex + Rh1 daily for 4 weeks. All the animals were killed by decapitation 12 h after the last injection. Approximately 0.8 mL trunk blood was collected into centrifuge tubes containing 100 *µ*L 0.3 M EDTA for radioimmunoassay. Collected blood was immediately centrifuged by using a Sorvall RC-3 centrifuge at 3000 g for 15 min. The supernatant was harvested and stored at −70°C for future use.

### 2.3. The Weight Ratio of the Spleen in Mice

The weight ratio of spleen is calculated according to the following formula: weight of an organ (mg)/body weight of a mouse (g) × 100%.

### 2.4. Measurement of Antibodies

Serum levels of antibodies reactive to double stranded DNA (dsDNA), antinuclear antibody (ANA), and antimitochondrial antibody (AMA) were measured by ELISA, as described previously [14].

### 2.5. Assessment of Proteinuria

To test the urinary protein excretion, mice were placed in metabolic cages after the last administration and urine samples were collected over a 24 h period. Protein concentrations were determined using urine protein test kit (Nanjing Jiancheng Bioengineering Institute, Nanjing, China), according to the manufacturer's instructions.

### 2.6. Isolation of Splenic T Cells

Mouse spleens were harvested and washed twice with ice-cold phosphate-buffered saline (PBS). Spleens were grinded in cold PBS containing 2% FBS and filtered with a 200 mesh strainer. Cells were washed in PBS containing 2% FBS by centrifugation at 1200 rpm at 4°C for 10 min and the supernatants were discarded. After resuspension in 1 mL PBS containing 2% FBS, 100 *μ*L aliquots of cell suspension were added to the appropriate tubes. Monoclonal antibodies (CD3-FITC, CD8-PerCP, and CD4-PE, 10 *μ*L for each) were added along with appropriate isotypic negative. These tubes were incubated for 10 min at 4°C. Then 0.5 mL Red Blood Cell Lysis Buffer (R&D systems, Minneapolis, MN, USA) was added in the tubes and the cells were centrifuged at 1200 rpm for 10 min. The cells were washed with 1 mL PBS, centrifuged at rpm for 10 min, and resuspended in 0.2 mL PBS. For each sample, 10000 events were analyzed and the proportions of CD3+CD4+ and CD3+CD8+ splenic lymphocytes were determined by flow cytometry and CD3+CD4+ T cells were isolated for the next experiments. The ratio of CD4+/CD8+ T cells was then calculated.

### 2.7. T Cell Proliferation Assay

CD3+CD4+ T cells were isolated by flow cytometry and then suspended in RPMI 1640 culture medium and placed in 96-well round-bottom plates at a cell density of 2 × 10^6^/mL to proliferate. RPMI-1640 medium with or without concanavalin A (Con A, 5 mg/L, Sigma, St. Louis, Mo, USA) was added and incubated at 37°C under 5% CO_2_ for 72 h. Then, 10 *μ*L 3-(4,5-dimethyl-2-thiazolyl)-2,5-diphenyl-2-H-tetrazolium bromide (MTT, 5 mg/mL) was added to each well, and then plate was incubated for another 4 h. Finally, 100 *μ*L SDS-isobutanol-HCl solution (10% SDS, 5% isobutanol, and 12 Mm HCl) was added to dissolve the MTT crystals [15, 16]. The optical density was measured with a microplate reader at the wavelength of 570 nm.

### 2.8. Real-Time RT-PCR Analysis of Cytokine mRNA in CD4+ Spleen Cells

Real-time RT-PCR was performed as described previously [17, 18]. Primers used for each cytokine are listed as follows: IFN-*γ*, forward: 5′-GTC AAC AAC CCA CAG GTC CA-3′; reverse: 5′-CGA CTC CTT TTC CGC TTC CT-3′; IL-4, forward: 5′-ATG GAT GTG CCA AAC GTC CT-3′; reverse: 5′-AAG CAC CTT GGA AGC CCT AC-3′; *β*-actin, forward: 5′-CCT CTA TGC CAA CAC AGT GC-3′; reverse: 5′-GTA CTC CTG CTT GCT GAT CC-3′. The relative expression of mRNA in each sample was normalized to its *β*-actin. The relative expression of mRNA was calculated as 2^−ΔΔCt^.

### 2.9. Statistical Analysis

All data were presented as the means ± standard deviation (S.D). Statistical significance was determined using SPSS 18.0 for Windows. Data analysis was performed by one-way analysis of variance (ANOVA), followed by Fisher's LSD. Differences with *P* values < 0.05 were considered to be statistically significant.

## 3. Results

### 3.1. Rh1 Increased the Spleen/Weight Ratio of MRL/lpr Mice

**Figure 1:**
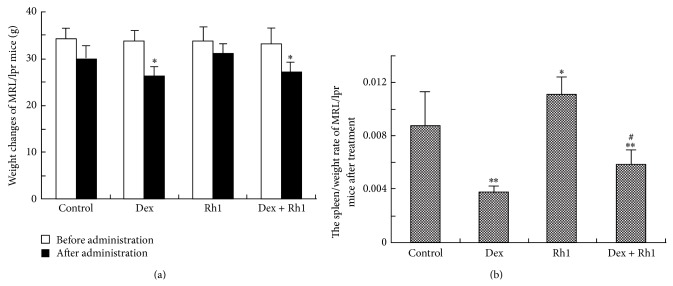
The effect of Dex in combination with Rh1 on the body weight and spleen/weight ratio of MRL/lpr mice. (a) The body weight changes MRL/lpr mice before and after treatment; (b) the spleen/weight ratio of MRL/lpr mice after 4-week treatment. Each bar represents mean ± S.D. (*n* = 6). ^∗^ *P* < 0.05, ^∗∗^ *P* < 0.01, compared with control; ^#^ *P* < 0.05, compared with Dex group.

Compared with MRL/lpr mice, the body weights of Dex- and Dex + Rh1-treated mice were significantly decreased (Figure 1(a)). Rh1 alone showed little effect on the mice body weight compared with control group. There were no significant differences in the body weight between Dex and Dex + Rh1 groups (Figure 1(a)). The spleen/weight ratio in Dex group was significantly decreased compared with that in control group (Figure 1(b)). In the Rh1 group, the spleen/weight ratio was significantly increased compared with control group. The spleen/weight ratio of Dex + Rh1-treated mice was lower than that of MRL/lpr mice, but higher than that of Dex-treated mice.

### 3.2. Rh1 Enhanced the Effect of Dex on the Levels of Proteinuria, Anti-dsDNA, and Anti-ANA Antibodies in MRL/lpr Mice

**Figure 2:**
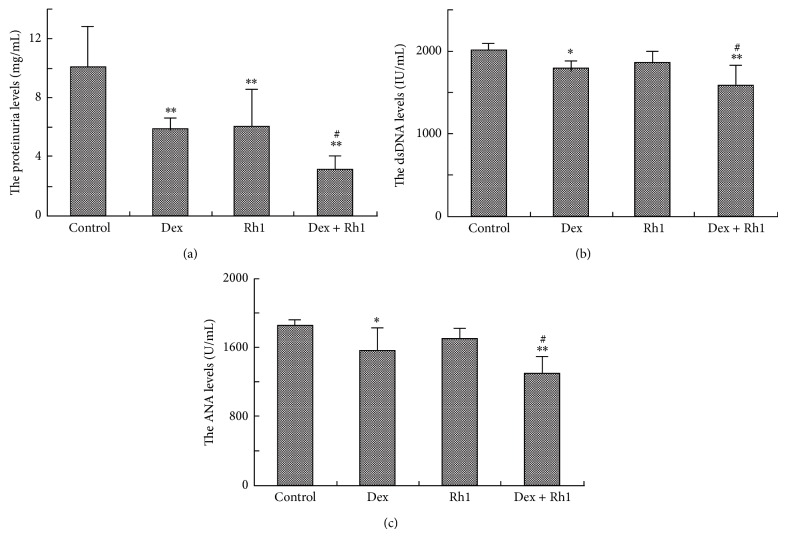
Rh1 improved the effect of Dex on proteinuria and anti-dsDNA and anti-ANA antibodies of MRL/lpr mice. (a) The proteinuria levels of MRL/lpr mice after treatment; (b) the anti-dsDNA levels of MRL/lpr mice after treatment; (c) the anti-ANA levels of MRL/lpr mice after treatment. Each bar represents mean ± S.D. (*n* = 6). ^∗^ *P* < 0.05, ^∗∗^ *P* < 0.01, compared with control; ^#^ *P* < 0.05, compared with Dex group.

To determine the therapeutic effect of Dex, Rh1, or Dex + Rh1 on MRL/lpr mice, we evaluated whether treatment with Dex, Rh1, or Dex + Rh1 improved the proteinuria and autoantibody production. Both Dex and Rh1 treatment significantly reduced the proteinuria levels (Figure 2(a)). The proteinuria level in Dex + Rh1 group was further decreased compared with that in Dex group. Compared with MRL/lpr mice, the levels of anti-dsDNA and anti-ANA autoantibodies in Dex-treated mice were significantly decreased (Figures 2(b) and 2(c)). In the Dex + Rh1 group, the levels of anti-dsDNA and anti-ANA autoantibodies were further downregulated compared with those of Dex group. Although the anti-dsDNA and anti-ANA levels were lower in Rh1 group than in control group, the differences between the two groups were not significant.

### 3.3. Proportion of CD3+CD4+ and CD3+CD8+ Splenic Lymphocytes

**Figure 3:**
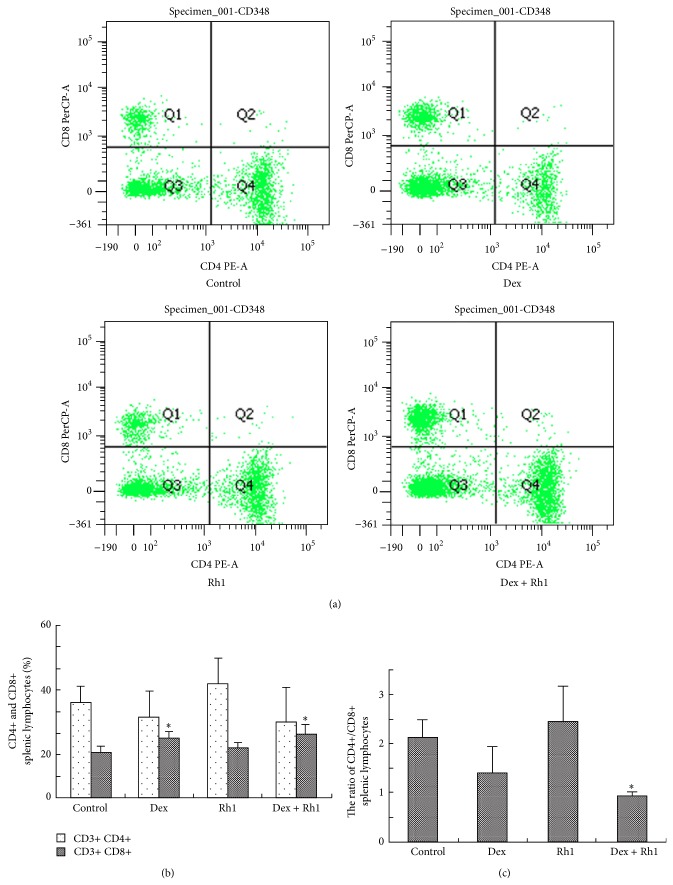
The proportion of CD3+CD4+ and CD3+CD8+ splenic lymphocytes after treatment. (a) Analysis of CD3+CD4+ and CD3+CD8+ splenic lymphocytes. Splenic lymphocytes were obtained from 3 mice randomly selected from each group. Splenic lymphocytes were stained by monoclonal antibodies against CD3, CD8, and CD4 and then analyzed by flow cytometry; (b) the proportion of CD3+CD4+ and CD3+CD8+ splenic lymphocytes; (c) the ratio of CD4+/CD8+ splenic lymphocytes. Each bar represents mean ± S.D. (*n* = 3). ^∗^ *P* < 0.05, compared with control.

CD3+CD4+ T cells are required for the development of human LE-like disease, and anti-CD4 antibody therapy decreases lymphoproliferation and autoantibody production and protects renal functions in MRL/lpr mice [19–21]. Therefore, we further examined the effect of Rh1 in combination with Dex on CD4+ and CD8+ splenic lymphocytes. After 4-week treatment, the proportion of CD4+ splenic lymphocytes was not significantly altered by Dex, Rh1, or Dex + Rh1 compared with control (Figure 3(b)). Meanwhile, the proportion of CD8+ splenic lymphocytes was significantly increased in Dex and Dex + Rh1 groups compared with control (Figure 3(b)). But there was no significant difference between Dex group and Dex + Rh1 group. As shown in Figure 3(c), the ratio of CD4+/CD8+ splenic lymphocytes was 2.12 ± 0.34 in control group, 1.38 ± 0.53 in Dex group, 2.43 ± 0.69 in Rh1 group, and 0.96 ± 0.06 in Dex + Rh1 group. There was a significant difference between control group and Dex + Rh1 group. However, no significant difference was determined between Dex group and control group and between Dex group and Dex + Rh1 group.

### 3.4. Dex Increased Con A-Induced Proliferation of CD4+ Splenic Lymphocytes

**Figure 4:**
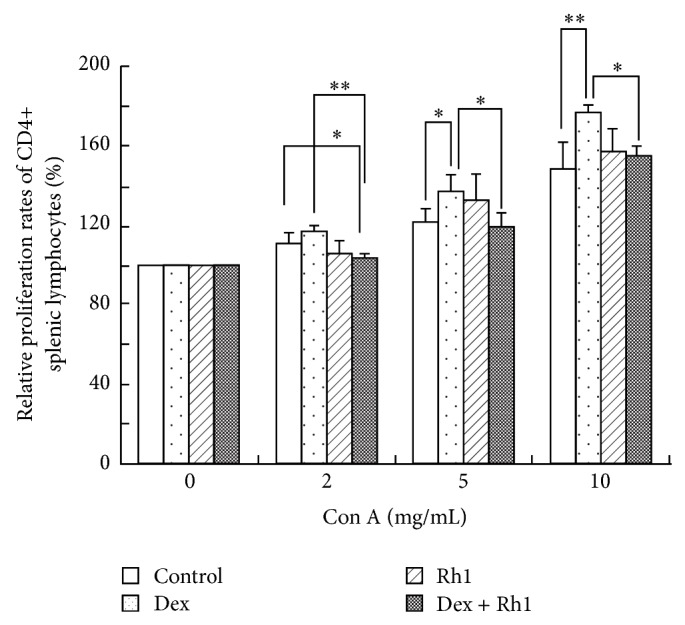
Proliferation of CD4+ splenic lymphocytes. CD4+ splenic lymphocytes isolated from mice spleen were stimulated with indicated concentrations of Con A for 72 h. MTT assay was performed to determine the proliferation of cells. Each bar represents mean ± S.D. (*n* = 3). ^∗^ *P* < 0.05, ^∗∗^ *P* < 0.01.

As shown by previous study [22], CD4+ splenic lymphocytes isolated from control mice spleen displayed a deficient proliferative response to Con A (Figure 4). Dex treatment increased splenic lymphocytes proliferation in response to various concentrations of Con A. However, the proliferation of splenic lymphocytes in response to various concentrations of Con A was decreased in Rh1 + Dex group compared with Dex group.

### 3.5. Expression of Th1/Th2 Cytokine mRNA in CD4+ Splenic Lymphocytes

**Figure 5:**
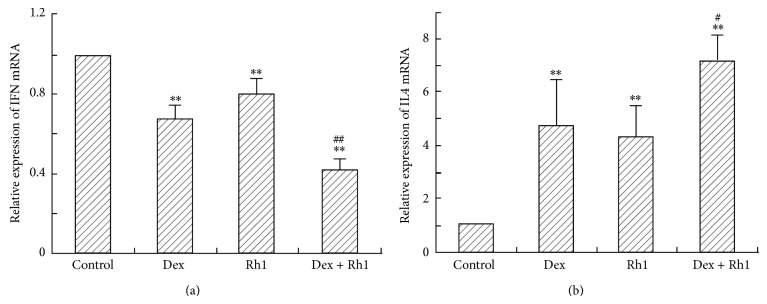
Expression of IFN-*γ* and IL-4 mRNA in the CD4+ splenic lymphocytes. Total RNA was isolated from CD4+ splenic lymphocytes and then real-time RT-PCR was performed to determine the level of IFN-*γ* and IL-4 mRNA. The expression of IFN-*γ* and IL-4 mRNA was normalized by *β*-actin. Each bar represents mean ± S.D. (*n* = 3). ^∗∗^ *P* < 0.01, compared with control; ^#^ *P* < 0.05, ^##^ *P* < 0.01, compared with Dex group.

IFN-*γ* and IL-4 are major mediators of several autoimmune and inflammatory diseases. Therefore, we determined the mRNA levels of IFN-*γ* and IL-4 in CD4+ splenic lymphocytes from MRL/lpr mice that were treated with Dex, Rh1, or Dex + Rh1. As shown in Figure 5(a), the expression of IFN-*γ* mRNA was suppressed by Dex and Rh1 compared with control. In the Dex + Rh1 group, the IFN-*γ* mRNA level was further decreased compared with Dex group. In contrast, the IL-4 mRNA was increased by Dex and Rh1 compared with control (Figure 5(b)). Rh1 in combination with Dex induced a further increase of IL-4 mRNA level compared with Dex alone.

## 4. Discussion

MRL/lpr mice develop a systemic autoimmune disease similar to SLE in humans. Although GCs and several immunosuppressive agents can effectively control the disease activities, patients may also suffer from serious adverse effects caused by the treatment agents. In the present study, our data demonstrated that Rh1 is able to enhance the ability of Dex on the reduction of proteinuria and autoantibodies in MRL/lpr mice. After 4-week treatment, Dex combined with Rh1 increased the proportion of CD8+ splenic lymphocytes and decreased the ratio of CD4+/CD8+ splenic lymphocytes, whereas it showed little effect on the proportion of CD4+ splenic lymphocytes. Con A-induced proliferation of CD4+ splenic lymphocytes was increased by Dex, whereas it is suppressed by Rh1 cotreatment. Rh1 also enhanced the ability of Dex on the reduction of IFN-*γ* mRNA and the increase of IL-4 mRNA.

It has been reported that elevated GCs levels induce spleen lymphocyte apoptosis, which may account for lymphocytes reduction and spleen atrophy [23]. GCs evoke apoptosis through regulating Bax and Bcl-2 mRNA and proteins expression after activating glucocorticoids receptor (GR) [24]. Our results showed that Dex treatment markedly decreased the spleen spleen/weight ratio, whereas spleen/weight ratio of Dex + Rh1-treated mice was higher than that of Dex-treated mice, suggesting that cotreatment with Rh1 could prevent the reduction of spleen lymphocyte number and spleen atrophy induced by Dex.

In clinical practice, an insufficient response of an SLE patient to a given dose of GCs (GC-resistance) leads to prescription of a higher dose (>30 mg/day) or additional immunosuppressive drugs. Furthermore, it was reported that approximately 30% of patients failed to respond to even high doses of glucocorticoids [1, 2]. Serious side effects will also present in those patients who take great a dosage of GC for long time. The downregulation of GC on GR is one of the reasons of GC resistance and could therefore seriously decrease the efficacy of GC used clinically [13]. Therefore, it is critical to the development of newer more effective therapies for these difficult to manage disease conditions.

SLE is mainly characterized by the abnormal increase of autoantibodies [25]. The anti-dsDNA is usually used as a biomarker for the diagnosis of SLE and evaluation of its activity in patients [26–28]. The presence of ANA, found in 95% of SLE patients, is also a prevalent feature of SLE that plays a pivotal role in the disease pathogenesis [29]. The level of ANA is positively correlated with anti-dsDNA [30, 31]. As expected, Dex-treated MRL/lpr mice showed a remarkable decrease in anti-dsDNA and anti-ANA autoantibodies. The effect of Dex on autoantibodies was strengthened by Rh1. However, Rh1 alone showed little effect on these autoantibodies. Lupus nephritis is one of the most severe symptoms related to the cause of death in human SLE. Our study also demonstrated that the level of proteinuria was further decreased by Dex combined with Rh1 compared with Dex alone. Therefore, our data suggest that Rh1 may be also able to potentiate the effect of Dex in the treatment of SLE.

Numerous studies indicate that the induction of anti-dsDNA autoantibodies in SLE are antigen-driven and thus T cell dependent response [32]. Koh et al. [33] showed that the disease was alleviated with a reduction in autoantibody production in CD4−/− lpr mice compared to littermate controls. The disease was also improved by T cell depletion and by combined anti-CD4 and anti-CD8 mAb therapy but not by anti-CD8 mAb therapy alone [34], indicating that SLE is dependent on CD4+ T cells. In the present study, our data showed that both Dex and Rh1 did not change the proportion of CD4+ T cells in spleen, whereas they increased the proportion of CD8+ T cells. Furthermore, Dex in combination with Rh1 decreased the ratio of CD4+/CD8+ splenic lymphocytes. Moreover, the proliferation of CD4+ splenic lymphocytes isolated from Dex-treated MRL/lpr mice after Con A stimulation was speedy compared with the cells obtained from control MRL/lpr mice spleen. However, Rh1 cotreatment with Dex suppressed the proliferation of CD4+ splenic lymphocytes. Our data reveal that Rh1 may inhibit the activation of CD4+ cells induced by Dex treatment.

Disturbed expression of Th1/Th2 cytokines is suggested to contribute to the pathogenesis of SLE [35, 36]. Th1- and Th2-cells are able to express their key cytokines IFN-*γ* and IL-4, respectively. In the peripheral blood mononuclear cells (PBMC) from SLE patients, the expression of IFN-*γ* and IL-10 mRNA is increased and IL-4 mRNA is decreased [37]. Sugimoto et al. [38] showed that the number of CD4+ T cells producing IFN-*γ* was increased, and the number of CD4+ T cells producing IL-4 was significantly decreased in SLE patients. In the current study, our study showed that the expression of Th1 cytokine IFN-*γ* mRNA was reduced in Dex-treated mice. However, the IL-4 mRNA level was increased in the CD4+ splenic lymphocytes isolated from Dex-treated MRL/lpr mice. The effect of Dex on IFN-*γ* and IL-4 mRNA expression was further enhanced by the cotreatment of Rh1. It is widely recognized that GCs are able to inhibit the expression of IFN-*γ* in MRL/lpr mice and SLE patients. However, the results about GCs on IL-4 production by T cells are inconsistent [39, 40]. GCs were reported to increase the production of IL-4 by mouse T cells, whereas they suppressed the production of IL-4 by human lymphocytes [39, 40]. GCs also inhibit the serum levels of IL-4 and the mRNA expression in human peripheral blood CD4+ T cells [41, 42]. However, pretreatment with Dex increased IL-2 induced IL-4 production in rat CD4+ T cells, whereas it decreased the synthesis of IFN-*γ* [43]. Dex decreased IFN-*γ* production and increased IL-4 and IL-10 production by tetanus-stimulated human PBMC [44]. These diverse results may be due to the differences in cell types, species, or the treatment methods. In our study, we showed that Dex inhibited IFN-*γ* production, while increasing IL-4 level, indicating GCs may induce a shift from the Th1 to Th2 profile of cytokine secretion, which may contribute to the mechanisms for GCs treatment.

Taken together, our study demonstrated that Rh1 may enhance the effect of Dex in the treatment of experimental SLE mice. Rh1 combined with Dex decreased the ratio of CD4+/CD8+ splenic lymphocytes but did not alter the proportion of CD4+ splenic lymphocytes. Rh1 combined with Dex also suppressed Con A-induced proliferation of CD4+ splenic lymphocytes and regulated the expression of Th1/Th2 cytokines. These results suggest that regulating CD4+ T cells activation and the ratio of Th1/Th2 cytokines may contribute to the enhancement of Dex on MRL/lpr mice. However, whether the Rh1 could also exhibit similar effect in humans needs to be further studied.
